# A Spatial-Spectral Classification Method Based on Deep Learning for Controlling Pelagic Fish Landings in Chile

**DOI:** 10.3390/s23218909

**Published:** 2023-11-02

**Authors:** Jorge E. Pezoa, Diego A. Ramírez, Cristofher A. Godoy, María F. Saavedra, Silvia E. Restrepo, Pablo A. Coelho-Caro, Christopher A. Flores, Francisco G. Pérez, Sergio N. Torres, Mauricio A. Urbina

**Affiliations:** 1 Department of Electrical Engineering, Universidad de Concepción, Concepción 4070409, Chile; 2 Department of Zoology, Universidad de Concepción, Concepción 4070409, Chile; 3 Department of Electrical Engineering, Universidad Católica de la Santísima Concepción, Concepción 4090541, Chile; 4 Centro de Energía, Universidad Católica de la Santísima Concepción, Concepción 4090541, Chile; 5 School of Engineering, Architecture and Design, Universidad San Sebastián, Concepción 4080871, Chile; 6 Institute of Engineering Sciences, Universidad de O’Higgins, Rancagua 2841959, Chile; 7 Instituto Milenio de Oceanografía (IMO), Universidad de Concepción, Concepción 4070409, Chile

**Keywords:** deep learning, fish, hyperspectral imaging, image processing, machine learning, VIS-NIR

## Abstract

Fishing has provided mankind with a protein-rich source of food and labor, allowing for the development of an important industry, which has led to the overexploitation of most targeted fish species. The sustainable management of these natural resources requires effective control of fish landings and, therefore, an accurate calculation of fishing quotas. This work proposes a deep learning-based spatial-spectral method to classify five pelagic species of interest for the Chilean fishing industry, including the targeted *Engraulis ringens, Merluccius gayi*, and *Strangomera bentincki* and non-targeted *Normanichthtys crockeri* and *Stromateus stellatus* fish species. This proof-of-concept method is composed of two channels of a convolutional neural network (CNN) architecture that processes the Red–Green–Blue (*RGB*) images and the visible and near-infrared (VIS-NIR) reflectance spectra of each species. The classification results of the CNN model achieved over 94% in all performance metrics, outperforming other state-of-the-art techniques. These results support the potential use of the proposed method to automatically monitor fish landings and, therefore, ensure compliance with the established fishing quotas.

## 1. Introduction

Fish have long been a crucial source of protein for human consumption. Unfortunately, poor management has led to the overexploitation of most targeted fish species and endangered the remaining ones [1]. The last monitoring report of the Food and Agriculture Organization (FAO) stated that, in the last 40 years, the percentage of marine fish stocks fished within biologically sustainable levels has decreased by 25%, while stocks fished at biologically unsustainable levels have increased by 20% [2]. Furthermore, it is recognized that 33% of fish stocks that are currently being overfished will not be recovered in the short term [2]. In Chile, as in many developing and underdeveloped countries, the overfishing problem is exacerbated by illegal fishing and the limited control of fish landings due to a lack of manpower and technology [3].

The sustainable management of fishing stocks has proved to be challenging, and it is a multivariate problem [4]. Researchers and the FAO agree that in order to achieve sustainable fishing, developed countries must support developing nations in policymaking and coordination and in deploying advanced management and control technologies [2,5]. Countries have made progress conserving and managing fish stocks; however, the current control measures for fish exploitation are input and out controls. Input control mechanisms take the form of restrictions or closures of areas, while output controls are typically enforced as catch quotas [4]. This paper focuses on contributing to the catch quota control stage to provide a modern digital classification method.

One of the underlying problems of controlling catch quotas is the accurate identification of the fish landed [6]. Without a fishing officer, by-catch is usually reported instead of the under-quote specie. This leads to underestimating the targeted fish quota and, therefore, imprecise biomass estimations [4]. Fish identification is challenging due to the diversity of fish species and the large volume of fish landed. The Chilean authority, Servicio Nacional de Pesca y Acuicultura (SERNAPESCA), is not capable of monitoring more than 5% of the fish landed (by biomass), and no more than 20% of the landings are inspected on-site [7]. Thus, the remaining uncontrolled volume must be reported by fishermen. SERNAPESCA argues that the actual fishing is misreported and estimates that approximately 60% of the reported volume corresponds to illegal fishing [7]. Second, during catch controls, the species classification is carried out by officers using “folk taxonomy”, i.e., officers judge similarities among species based on sight and experience [8]. Therefore, classification relies heavily on the level of expertise of the officers. Further errors are introduced, such as biases, calculations, and transcription errors, as they are typed by hand and in situ. Furthermore, the limited evidence could be further questioned by the fisherman. All of these error sources have had a negative impact on the control measures taken by authorities for managing fisheries.

Because the control process of pelagic fish species in Chile uses no digital technology at all, SERNAPESCA states in its strategic roadmap that introducing digital technology would greatly assist their efforts for the recovery and management of threatened and endangered fish stocks [9,10].

Based on the above, this paper presents a Deep Learning (DL)-based classification method for automatically controlling catch quotas in Chilean pelagic fisheries. This method is a proof-of-concept to deliver a future production method for assisting SERNAPESCA’s catch control processes. The proposed method classifies five different pelagic fish species, differing in size, shape, and color, but all of which are of economic interest for both the Chilean authorities and the fishing industry: *Engraulis ringens, Merluccius gayi, Normanichthtys crockeri, Stromateus stellatus,* and *Strangomera bentincki* [11].

We hypothesize that spectral reflectance signatures are species-specific based on external differences in shape, color, scales, and mucus (spatial and structural morphology). Therefore, they could be used to develop a highly accurate discrimination method. The necessity of including spectral signatures and developing fish classification systems incorporating spectral information arises from many fish species having similar physical appearance in terms of size, shape, and texture. We collected Hyperspectral (HS) cubes in the Visible and Near-Infrared (VIS-NIR) spectral bands and created a database containing 5000 images. The hypercubes were processed and labeled to render Red–Green–Blue (*RGB*) images and reflectance spectra for each species. We then designed a novel two-channel DL architecture to classify pelagic fish species. The first channel, termed “the spatial channel”, uses a four-layer, two-dimensional Convolutional Neural Network (CNN) to analyze the *RGB* images and extracts spatial classification features. The second channel, termed “the spectral channel”, uses a four-layer, one-dimensional CNN and processes averaged reflectance spectra to extract wavelength-dependent classification features. Next, the designed architecture combines the features from each channel using dimension reduction techniques and ultimately produces a classification function. The DL-based classification method in a laboratory environment with controlled illumination conditions achieved an average classification Accuracy (ACC) of 94.26%. For comparison, we also developed classifiers based on state-of-the-art techniques such as using Support Vector Machine (SVM), Histogram of Oriented Gradients (HOG)-based features, and spatial (pixels-based features) and spectral information. These models were outperformed by the DL-based spatial-spectral classification method, with values being between 6% and 36% higher when the average classification ACC was used as a comparison metric. These results show the ability of the CNN to extract representative patterns from the images, especially when considering the spectral information of the fish, thus allowing us to discriminate between species that may be physically similar but belong to different classes. Taking the above into account, the main contributions of this study are:

- A database of spectral signatures of several fish species;
- A spatial-spectral classification method for the automatic identification of pelagic species

The rest of this paper is organized as follows. Section 2 briefly explores related studies. Section 3 describes the fish sample preparation and main characteristics of the hyperspectral imaging system, the pre-processing of information gathered by the acquisition system, and the design of the classifiers of fish species. Section 4 presents the results of the classification method. Finally, Section 5 states the main conclusions and delineates future directions.

## 2. Related Work

The most relevant scientific and technical work directly related to this paper has been compiled in [12].

The automatic classification of fish species has been carried out, depending on the application’s needs, under special laboratory conditions or in the fish’s natural environment. In the laboratory, analyses of fish behavior for certain species have been reported; in the field, techniques for estimating fish quotas have been investigated [13,14]. Spampinato et al. proposed a classification system for recognizing a wide variety of deep-sea fish species [15]. The classification system used a combination of morphological characteristics, such as shape and texture. These features were engineered to be invariant to transformations. Images were then taken in the natural environment of the species of interest, wherein the swimming trajectories were extracted and studied to analyze the behavior of the fish species. Hu et al. presented a novel method for classifying fish species based on color and texture characteristics [16]. By employing an SVM technique, they remotely classified species and reported fish diseases in rural aquaculture farms in China. Interestingly, fish images were acquired using smartphones, and data processing was conducted at remote centers in a manner resembling a cloud computing environment.

White et al. proposed a computer vision method sped up using specialized hardware to distinguish certain fish species and determine their length in real time [17]. To accomplish this, fish orientations had to be determined first with high precision by using the method of the moments. Next, species were identified using morphological characteristics, such as flat or round shapes. Pixel calibration was used to compute the length of each fish. Storbeck and Daan developed a fish species recognition system based on computer vision techniques and neural network models [18]. The vision system measured several fish features, including the width and height at various locations along the fish bodies. Such a system could distinguish samples of new fish species moving on a conveyor belt.

Most vision systems have so far focused on the visible part of the electromagnetic spectrum. These systems use off-the-shelf digital cameras to acquire images, sometimes neglecting the characteristics of the fish that can be evaluated in other spectral bands. We claim that the new Hyperspectral Imaging (HSI) techniques, which combine traditional optical spectroscopy and computer vision, may obtain valuable spectral and spatial information, allowing for accurate fish species discrimination [19,20]. To the best of our knowledge, some recently developed applications in aquaculture and fishing using HSI are related to the analysis and evaluation of fish quality. Among them, we have found in the literature parasite detection systems for measuring the physical properties of fish, as well as discrimination systems for fish freshness [20,21,22]. Our group has also developed fish classifiers based on SVM and Near-Infrared (NIR) HSI [23].

Novel data science techniques have also been used for fish classification. For example, from a database known as Fish4Knowledge, different works have been proposed for classifying fish species [24,25]. This database contains images of 20+ fish species inside an aquarium. Similar to the Fish4Knowledge database, the Nature Conservancy Fisheries Monitoring, in conjunction with the Kaggle community, have launched a contest to develop algorithms for automatically detecting and classifying sport fishing species such as albacore, bigeye tuna, yellowfin tuna, mahi-mahi, opah, sharks, and other species [26]. Due to the availability of these types of databases and the improvements in computational processing, algorithms based on DL have been developed to classify fish species. In this sense, algorithms based on CNNs allow for the automatic extraction of complex patterns from images, such as those based on appearance (textures and colors among others) and those based on geometry (length and fins among others) [27,28]. Some works have demonstrated that combining spatial and spectral information improves image classification tasks by finding relationships between the spectral signatures and pixels, even under high-dimension problems, and the improvement is accomplished using fewer training examples [29,30].

## 3. Materials and Methods

### 3.1. Sample Preparation: Pelagic Fish

Samples of five different pelagic fish species freshly caught by fishermen in the Bio-Bio region were provided by SERNAPESCA officers. Typically, fishing vessels left for fishing grounds just after midnight, hauling/trawling until early morning and returning to shore the following day. Fishing officers collected the pelagic species and delivered them to the Laboratorio de Fisiología Animal Comparada (Comparative Animal Physiology Lab) at the Universidad de Concecpión (UdeC). Our experiments considered freshly caught samples of the following Chilean pelagic species: *Engraulis ringens, Merluccius gayi, Normanichthys crockeri, Stromateus stellatus,* and *Strangomera bentincki*. Table 1 shows the number of samples per species.

Fish species were labeled, and the wet mass, length, species ID, and measurement day were recorded. Fish samples were gently washed with filtered (300 um) seawater to withdraw any residual impurities on the external surface, thus potentially altering skin hyperspectral behavior.

### 3.2. Hyperspectral Imaging Setup and Acquisition

**Figure 1:**
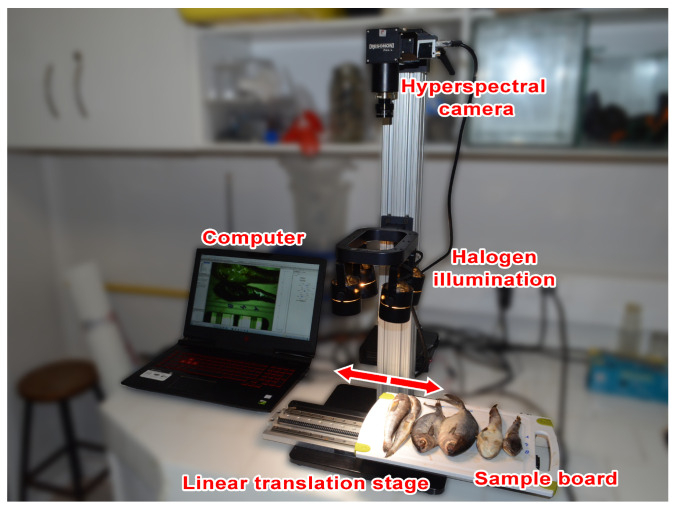
Setup of the hyperspectral imaging system for reflectance measurements. Note that the moving platform is indicated by the red arrows.

The fish samples were scanned by employing a RESONON Benchtop System for reflectance measurements, consisting of a hyperspectral camera, a 4-fixed halogen lighting assembly illuminating from the top, a horizontally moving platform, and a computer, as shown in Figure 1. A hyperspectral camera Pika L model with 281 VIS-NIR channels in the range of 400–1000 nm, 2.1 nm of spectral resolution (FWHM), 900 spatial pixels per line, and an acquisition speed of 100 frames per second was used. The hyperspectral camera and the translation stage were controlled using a laptop with proprietary software ( SpectrononPro Version 3.4.11, RESONON, Bozeman, MT, USA) to generate hyperspectral image cubes of each fish. The moving platform was set to scan fish samples at 1.55 cm/s.

Hyperspectral images were collected on the same day samples arrived, and only fish in good condition were used to collect HSI data. Fish missing scales, fins, or those having any external damage were excluded from the analysis. Fish samples were placed on a Teflon board that moved under the hyperspectral camera during scanning, as shown in Figure 1. The lighting assembly and hyperspectral camera were positioned at 22 cm and 59 cm above the sample board, respectively.

### 3.3. Data Prepossessing

Hyperspectral images are rich in spatial and spectral information that can be exploited by feeding them to a classification method. Our data show that each fish species exhibited a species-specific spectral and spatial signature, regardless of fish size within each species. The procedures used to generate spectral signatures and *RGB* images from the hyperspectral images were as follows.

#### 3.3.1. Extraction of Spectral Signatures

The collected fish hypercubes were calibrated to reflectance values in the range of [0,1] using a Spectralon^®^ diffuse reflectance standard with a typical reflectance value of 99%. The calibration procedure consists of capturing a hyperspectral frame of the reflectance standard and using it to normalize the intensities of the fish hyperspectral images. Let $A^{\left(\lambda \right)}\left(i,j\right)$ be the intensity (digital counts) of a fish hyperspectral image at the ij-th spatial pixel coordinates and wavelength λ; the corresponding calibrated reflectance hyperspectral image, $S^{\left(\lambda \right)}\left(i,j\right)$, is computed as:

(1) $$S^{\left(\lambda \right)}\left(i,j\right)=\frac{A^{\left(\lambda \right)}\left(i,j\right)}{R^{\left(\lambda \right)}},$$

where $R^{\left(\lambda \right)}$ is the intensity (digital counts) of the reflectance standard hyperspectral frame at wavelength λ.

**Figure 2:**
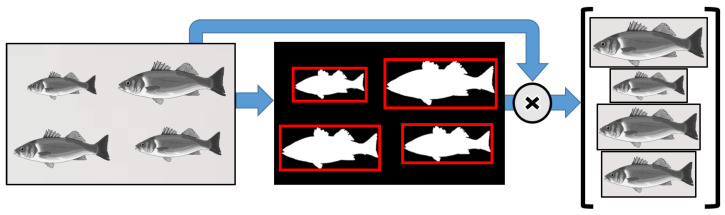
Sample separation from an image of a set of fish.

The calibrated hypercubes are then further processed to isolate every fish in the image via the 2-step segmentation procedure depicted in Figure 2. In this work, the whole fish RoI was employed for extracting the fish spectral reflectance features to ensure that the resulting reflectance curve captured all spectral information of each fish sample. First, at a high-contrast wavelength $\lambda_{s}$, a binary mask was generated for each fish in the image (the masks were constrained to red boxes); second, each single fish image was extracted by superimposing the masks onto the original image and cropping the area out of the box. This last step was then repeated through all the remaining wavelengths of the hypercube. The resulting hyperspectral fish images were then used to determine the reflectance curves of each fish.

The reflectance curve describes the percentage of light that the fish skin reflects at each wavelength. The reflectance curves represent the so-called spectral features or optical fingerprints specific to an object of interest.

**Figure 3:**
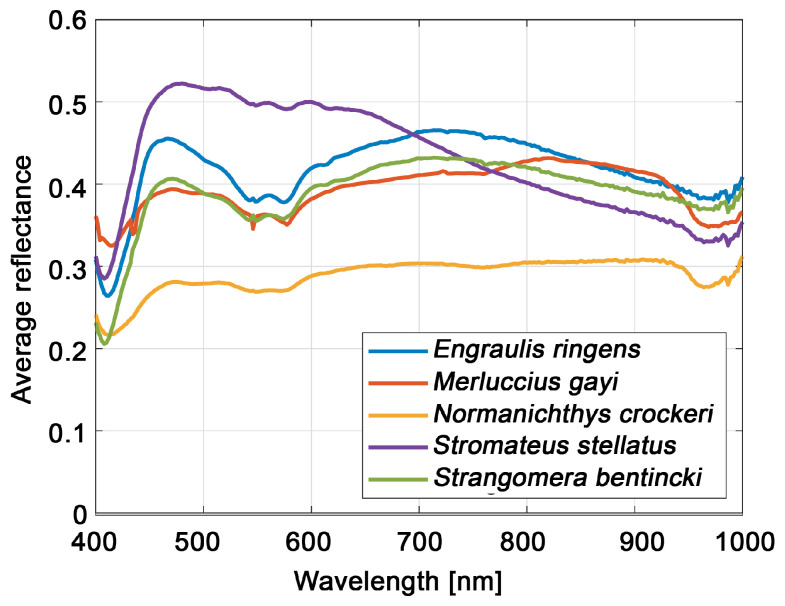
Whole-body average reflectance curves of the five species studied.

Let *K* be the total number of samples of a single fish species; then, the spatial average reflectance of the *k*-th fish sample is computed as:

(2) $${\bar{s}}_{k}\left(\lambda \right)=\frac{1}{N\times M}\sum_{i=1}^{N}\sum_{j=1}^{M}S_{k}\left(i,j,\lambda \right),$$

where $i=1,\ldots ,N,j=1,\ldots ,M,\lambda =\lambda_{1},\ldots ,\lambda_{L_{c}}$, and k=1,…,K. Figure 3 shows the average reflectance curves of the five species under study. These species-specific spectral signatures are considered a differentiating feature in training the classifiers. From examining Figure 3 it is intuitive to identify key features in the curves, such as valleys, peaks, slopes, and plateaus. It is important to indicate that we did not use the mean reflectance of the fish Region of Interest (RoI) to recognize species due to reflectance variation within fish samples belonging to the same species and the similar reflectance curves of different species.

**Figure 4:**
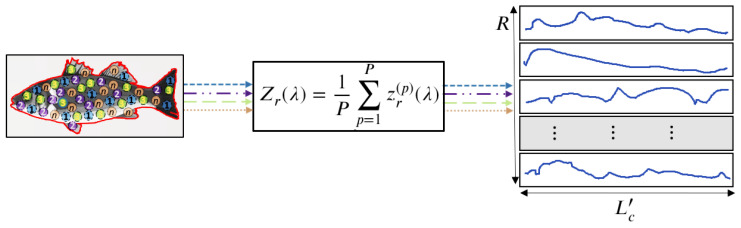
Extraction of average spectral signatures from a single fish hypercube. Note that the random points of the RoIs are indicated by numbers in the fish image.

To develop our classification models, we defined the spectral signatures for each species as the spatial average of the hyperspectral images over an RoI. Some pixels are randomly selected to define one or more RoIs in the segmented image, as depicted in Figure 4. The randomly generated RoIs are not necessarily contiguous. Let us decompose a single hypercube into *R* RoIs, with each one of them containing *P* pixels. Let $z_{r}^{\left(p\right)}\left(\lambda \right)$ be the reflectance of the *p*-th pixel in the *r*-th RoI. Then, the average spectral signature of the *r*-th RoI is computed as:

(3) $$Z_{r}\left(\lambda \right)=\frac{1}{P}\sum_{p=1}^{P}z_{r}^{\left(p\right)}\left(\lambda \right),$$

where r=1,…,R,p=1,…,P, and $\lambda =\lambda_{1},\ldots ,\lambda_{L_{c}^{\prime }}$. This procedure yields a set of *R* spectral signatures associated with the sample, and the spectral dimensions are reduced to $L_{c}^{\prime }\leq L_{c}$. Therefore, the spectral signatures of a single fish sample conform to an array of dimensions $R\times L_{c}^{\prime }$.

#### 3.3.2. Extraction of *RGB* Images

**Figure 5:**
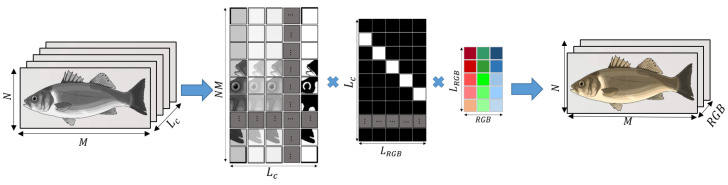
Generation of *RGB* images from the fish hypercubes.

Color *RGB* images were generated from the hyperspectral images by applying a method based on emulating the spectral sensitivity of an *RGB* camera, as illustrated in Figure 5. For this purpose, we employed the spectral sensitivity function of a Canon 1D Mark III camera as reported in the database provided by [31]. Particularly, the spectral sensitivity function of this camera model is described in 33 channels of 10 nm FWHM over the 400–720 nm range. In our *RGB* image extraction method, the relationship between the spectral sensitivity of the *RGB* camera and the *RGB* channels can be represented as a matrix with the dimensions $L_{RGB}\times RGB$, where $L_{RGB}$ corresponds to the spectral dimensions that integrate the 3 *RGB* channels and RGB represents the resulting red, green, and blue channels, respectively. Hyperspectral images were converted to *RGB* images as follows. First, the hyperspectral image of the dimensions $N\times M\times L_{c}$ is reordered into a matrix of dimensions $NM\times L_{c}$. Second, a projection matrix of dimensions $L_{c}\times L_{RGB}$ is employed to match the spectral dimensions of the hyperspectral system with the spectral dimensions of the *RGB* camera spectral sensitivity. This projection matrix averages the hyperspectral system output at all wavelengths $\lambda_{c}$ in the intervals $\left[\lambda_{RGB}\left(i\right),\lambda_{RGB}\left(i+1\right)\right]$ and is used to describe the spectral sensitivity of the *RGB* camera. In this manner, the projection matrix averages the hyperspectral channels $\lambda_{c}$ (281 channels, 2.1 nm FWHM) over the 33 channels (400–720 nm, 10 nm FWHM) of the *RGB* camera model. Finally, the *RGB* image is formed by (i) performing a matrix multiplication between the reordered hyperspectral image, the projection matrix, and the matrix of spectral sensitivity vs. *RGB*; and (ii) reordering the result. At the end of this stage, *RGB* images of 1070×260 pixels resolution in each R, G, and B channel were generated.

### 3.4. Design of Classifiers for Pelagic Fish Species

**Figure 6:**
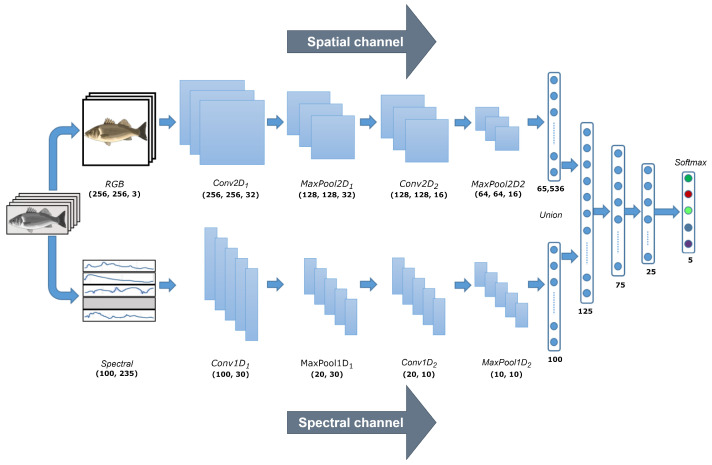
Architecture of CNN for the classification of pelagic species. The CNN has two input channels that independently extract features from the spatial and spectral channels (input data). The dimension flow of the CNN network is also provided in bold text.

A DL-based classification method was designed to automatically discriminate five different species of Chilean pelagic fishes. The *RGB* images and spectral signatures extracted from the hyperspectral images were input into a CNN. Three models of this network were evaluated, considering the following inputs: (i) *RGB* images; (ii) spectral signatures; and (iii) both *RGB* images and spectral signatures. In all cases, the network can automatically extract relevant features from the input data using convolution operations on the different input data. These features are used to classify the different fish species, as schematically shown in Figure 6. Specifically, we considered typical feature extraction steps for spatial and spectral channels composed of two convolutional layers, each of which were followed by an activation and pooling layer. We considered the widely used activation function Rectified Linear Unit (ReLU) to introduce nonlinearity and a max pooling method because it does not require tuning parameters. The spatial channel learns and automatically extracts morphological features from the fish *RGB* images, obtaining a vector of 65,536 features. In this sense, the initial CNN layers automatically allow for extraction of simple features (e.g., basic edges and shapes) from the *RGB* images, while the later layers learn complex features [32]. On the other hand, the spectral channel learns and automatically extracts attributes from the average reflectance spectra of the fish, resulting in a vector of 100 features. Finally, the features extracted from the two channels are combined in a regular dense multilayer neural network to perform the multi-class classification using a softmax function.

#### 3.4.1. Data Augmentation for Improving Classification

**Figure 7:**
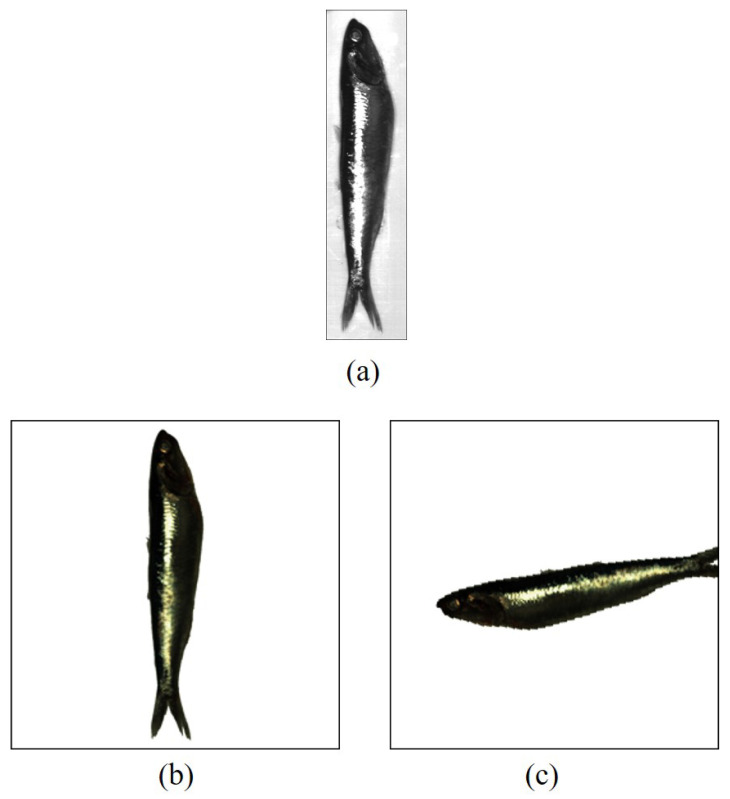
Example of image data augmentation for *Engraulis ringens*. (**a**) Original: 1070×260×3 pixels. (**b**) Rescaling: 256×256×3 pixels. (**c**) Final image: 256×256×3 pixels.

The training of a CNN requires many samples, which is difficult to achieve in practice. To overcome this limitation, we augmented the spectral data of each fish hypercube by extracting the reflectance curves of various random RoIs as described in Section 3.3.1. Also, we augmented the spatial data by randomly reorienting and rescaling the fish *RGB* images as described below. Note that the original *RGB* images, i.e., the result directly obtained by imaging the fish, have a dimension of 1070×260×3 pixels before rescaling (see Figure 7a).

Before augmenting the spatial data, the images are all rescaled to a size of 256×256 pixels, and the background is replaced by a white one. In this sense, we considered a typical value of 256 to reduce the dimensions of the original images, keeping a uniform size and reducing the model complexity for the training of the classifiers [33,34,35,36]. The fish was rotated, translated, and randomly flipped to perform data augmentation. Thus, we aimed to increase the number and variety of the samples, keeping the class distribution balanced [37,38]. Additionally, data augmentation allows models to learn the intrinsic features of the images, regardless of the transformations involved, for better model generalization [38,39].

Figure 7a shows an example image of *Engraulis ringens*, Figure 7b shows the resulting image after rescaling and background subtraction, and Figure 7c shows an example of an augmented image, which is acquired after performing a flip followed by a rotation and translation operation.

**Figure 8:**
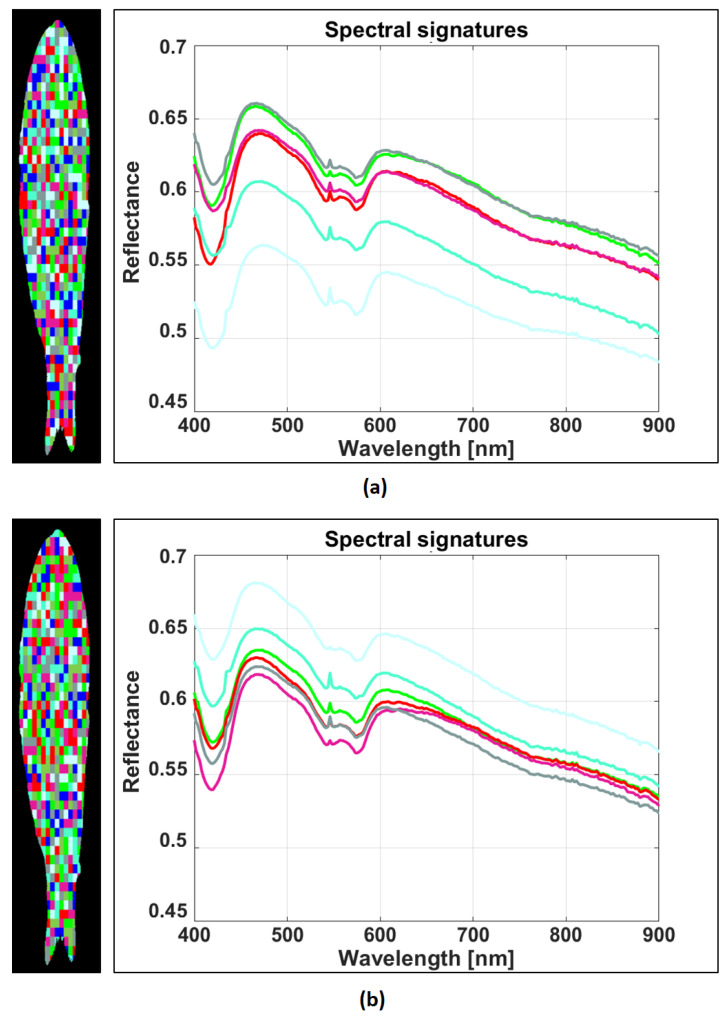
Example of spectral data augmentation for *Engraulis ringens*. (**a**,**b**) Spectral signatures (average reflectance) using two different sets of RoIs, respectively.

As mentioned above, the spectral signatures are augmented by generating different RoIs over an image to spatially average the reflectance associated with different zones of the fish. In this way, the resulting spectral signature is a good representation of the whole sample. Figure 8a,b show the spectral signatures of the same fish sample after generating two different sets of RoIs for performing the spatial averages.

Finally, at the end of this stage, a total number of 1000 images per class were obtained, as shown in Table 1.

#### 3.4.2. Convolutional Neural Network (CNN)

The proposed CNN automatically extracts key features from both the spatial (*RGB* images) and the spectral channel (curves of spectral signatures) independently, then concatenates these relevant features into a single feature vector that is used as an input to the classification stage, which is shown in Figure 6 [40]. Note that each input is evaluated independently and in combination, i.e., by using the spatial and spectral channels [41,42,43].

In a practical way, the network architecture is inspired by a simpler acquisition system composed of an *RGB* camera and a spectrometer, both of which deliver the input data to a classification method. This approach was employed because the hyperspectral data is large and redundant. By using this type of simpler architecture, the dimensions of the input data are reduced by the rescaling operations and extraction of spectral signatures from a subset of all available wavelengths. Also, this approach directly benefits the training of the CNN as the training time significantly decreases by reducing the dimensions of the input tensors. Each convolutional layer of the proposed architecture has a subsequent batch normalization stage and an ReLU activation layer. Also, after each MaxPooling stage, there are DropOut layers with a 0.25 probability [44]. After feature extraction, a fully connected network with a softmax layer was used to classify the images. Finally, we trained the network, considering the following hyperparameters: 4000 epochs, batch size = 32, 50 epochs for early stopping to avoid overfitting, considering 33% of the training data for validation, and Stochastic Gradient Descent (SGD) algorithm (learning rate = 0.05) [41,42,43]. Thus, the CNN network will evaluate and stop its training in a maximum of 4000 epochs if the validation error does not decrease in 50 consecutive epochs to avoid overfitting.

#### 3.4.3. Performance Evaluation

The training and testing of the classifiers were performed using a 5-fold cross-validation procedure to finally average the performance metrics [45]. During each of the five iterations of the cross-validation, 4000 data points were used to train the classifiers, while the remaining 1000 were used for performance evaluation purposes. Thus, it is possible to evaluate multiple times (k-fold) the generalization error of the classifiers on data not seen during the training stage [46]. This method also allows for a more detailed evaluation of the goodness-of-fit as opposed to a single evaluation, as when using the holdout technique (e.g., 66% for training and 34% for testing) [47]. The performance metrics selected for studying the classification results were Accuracy (ACC), Precision (PRE), Recall (REC), and *F*1-value (F1). These metrics are commonly used to assess classification algorithms’ performance. In this sense, Accuracy specifies the number of correctly classified examples, while Precision and Recall provide information on the number of positive predictions correctly classified and the number of positive cases that were classified, respectively [48]. In this regard, the F1 metric allows us to evaluate in a value the balance between Precision and Recall metrics, especially when multi-class problems are handled. Mathematically, ACC, PRE, REC, and F1 are defined as follows:

(4) $$\begin{array}{cc} ACC & =\frac{TP+TN}{TP+FP+TN+FN}, \end{array}$$

(5) $$\begin{array}{cc} PRE & =\frac{TP}{TP+FP}, \end{array}$$

(6) $$\begin{array}{cc} REC & =\frac{TP}{TP+FN}, \end{array}$$

(7) $$\begin{array}{cc} F1 & =\frac{2(PRE)(REC)}{PRE+REC}, \end{array}$$

where TP and TN are the True Positives and Negatives, respectively, while FP and FN are the False Positives and Negatives, respectively. Additionally, for each classifier, training and testing error curves were evaluated according to the performance in terms of the number of training examples and the Zero-One-Loss (*L*) metric between the predictions ($y_{i}$) and the actual classes ($y_{i}^{\prime }$) [49]:

(8) $$L\left(y_{i},y_{i}^{\prime }\right)=\left\{\begin{array}{cc} 1 & y_{i}\neq y_{i}^{\prime }\\ 0 & \mathrm{otherwise} \end{array}\right.$$

## 4. Results and Discussion

The performance of the proposed classification method was assessed by analyzing the impact that spectral and spatial features have on it and by comparing its performance against an SVM classification method. A linear kernel is considered to keep the rest of the parameters by default [50]. The performance of the SVM was studied using the total pixels of each *RGB* image and the features extracted using HOG. The most important parameters of this descriptor are the orientations, pixels per cell, and cells per block, which control the resulting number of extracted features. In this sense, the following parameters were considered: orientations = 8 (bins per histogram), pixels per cell = (16,16), and cells per block = (4,4) [51,52].

Table 2 shows the overall classification results of the CNN- and SVM-based algorithms on the test set. As noted, the performance of the CNN classification model that combines the *RGB* images and spectral signatures outperformed the others, obtaining values of over 94% in all performance metrics. In the case of SVM, using spectral information improved the performance of this classifier, especially when combined with HOG features.

**Figure 9:**
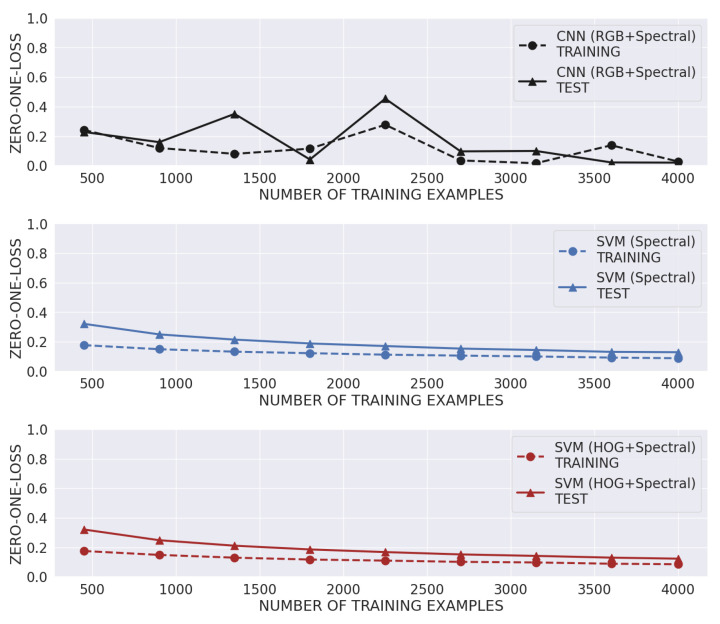
Comparative results of the training and test error for the best model using each feature type (input).

Figure 9 shows the training and test error as the number of training examples increases, considering the best models as indicated in Table 2. Thus, we aimed to evaluate the classifiers’ generalization error to determine if good training was performed, avoiding underfitting and overfitting problems; in other words, we assessed whether the models fitted too closely to the training data or if they were too simple [53]. As expected, it is possible to observe that in most cases, the training error curve is lower than the testing one. In this sense, no over-fitting tendency is observed in the classifiers as the curves decrease as the number of training samples increases. In the case of the best CNN model, although the training error curve increases around sample 2000, it then decreases towards the end of the curve.

**Figure 10:**
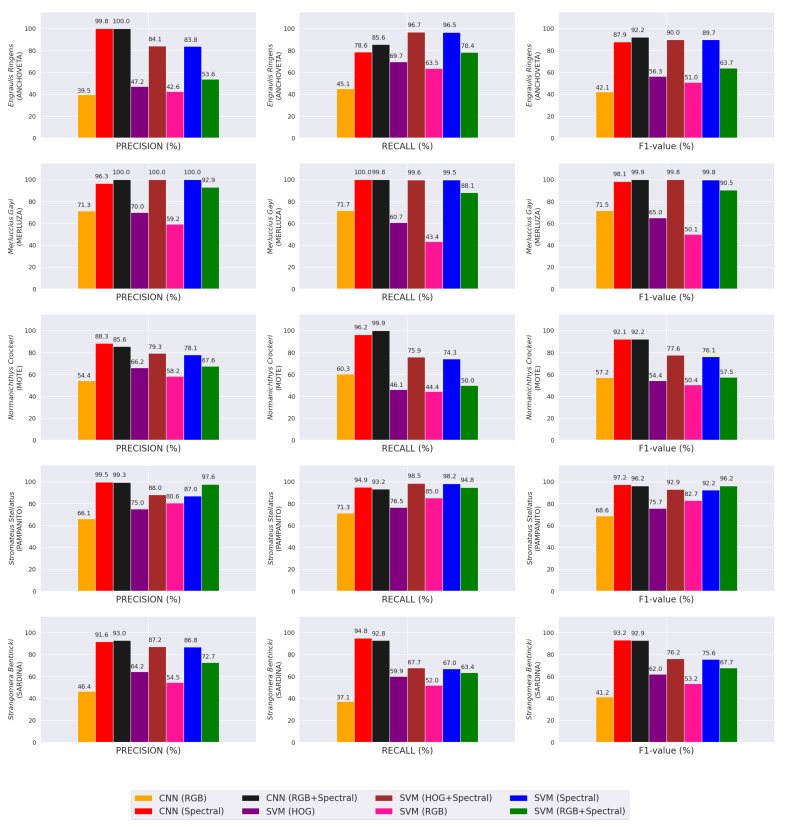
Comparative results for each problem class.

Figure 10 shows the classification results of the CNN- and SVM-based algorithms for each one of the five fish species. In most cases, the performance of CNN was better than the rest of the SVM-based algorithms, mainly when spectral signatures were used. The best classification performance reported by the proposed method was achieved for *Merluccius gayi*. This result could be attributed to its distinctive reflectance signature (refer to Figure 3) compared with the other fish species, especially at 600 [ nm].

## 5. Conclusions

A spatial-spectral method based on Deep Learning (DL) was developed and tested for the classification of five different pelagic fish species that are common and intensively fished by the Chilean fishing industry: *Engraulis ringens, Merluccius gayi, Normanichthys crockeri, Stromateus stellatus,* and *Strangomera bentincki*. The proposed method contributes to accurately and automatically controlling fishing quotas. Because many fish species have similar physical appearances (e.g., size, shape, and texture), we included the VIS-NIR reflectance signatures of the species for more accurate classification. Thus, we demonstrated the feasibility of using spectral information on fish species of comparable size and morphology.

Although we only used a total of 1000 images per species to generate the training and testing datasets in this work, the proposed method achieved accuracy rates of over 94%, outperforming the classifiers described as state of the art (refer to Table 2) with no problems of over-fitting because the training is stopped early if necessary (refer to Figure 9). This high performance was also seen in the classification results for each one of the classes under study (refer to Figure 10). The high performance levels show the feasibility of implementing this classification methodology in an industrial environment.

Future work from our research team will focus on refining our classifiers by acquiring more images of the pelagic species included in this study and other fish species (e.g., the Fish4Knowledge database) along the Chilean coast. Designing a model to estimate the size and weight of the landed fish is also desired, as it would contribute to better fish management; however, it is heavily conditioned to solid fish size–mass curves. The results show that developing a fully automated method capable of controlling fish landings in a real scenario is undoubtedly feasible and much needed under our current global over-fishing scenario.

## Figures and Tables

**Table 1:** The scientific names and number of samples for each pelagic fish species considered in this work.

Species	Image	Provided Samples	Augmented Samples	Number of Samples
*Engraulis ringens (Anchoveta)*	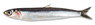	58	942	1000
*Merluccius gayi (Merluza)*	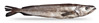	75	925	1000
*Normanichthys crockeri (Mote)*	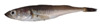	341	659	1000
*Stromateus stellatus (Pampanito)*	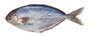	42	958	1000
*Strangomera bentincki (Sardina)*	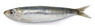	128	872	1000
	Total number of samples			5000

**Table 2:** Overall classification results.

Classifier	Accuracy (%)	Precision (%)	Recall (%)	F1-Value (%)
CNN (Spectral)	92.90	95.11	92.90	93.99
CNN (*RGB*)	57.10 *	55.53 *	57.10 *	56.30 *
CNN (*RGB* + Spectral)	94.26	95.58	94.26	94.92
SVM (Spectral)	87.10	87.13 *	87.10	87.11
SVM (*RGB*)	57.66 *	59.02 *	57.66 *	58.33 *
SVM (*RGB* + Spectral)	74.94 *	76.90 *	74.94 *	75.91 *
SVM (HOG)	62.58 *	64.52 *	62.58 *	63.54 *
SVM (HOG + Spectral)	87.68	87.71 *	87.68	87.69

* symbol denotes that statistically significant differences (*p* < 0.05) appeared between CNN (*RGB* + Spectral) and the rest of the models (Wilcoxon signed-rank test). In the case of SVM, *RGB* indicates using all pixels as features.

## Data Availability

The data presented in this study are available on request from the corresponding author. The data are not publicly available due to privacy reasons. The source code of this study is available at https://github.com/christopherfj/FishClassification_Sensors2023 (accessed on 26 August 2023).

## Abbreviations

The following abbreviations are used in this manuscript:

CNN	Convolutional Neural Network
DL	Deep Learning
FAO	Food and Agriculture Organization
HOG	Histogram of Oriented Gradient
HS	Hyperspectral
HSI	Hyperspectral Imaging
NIR	Near-Infrared
*RGB*	Red–Green–Blue
SERNAPESCA	Servicio Nacional de Pesca y Acuicultura
SVM	Support Vector Machine
VIS-NIR	Visible and Near-Infrared
HOG	Histogram of Oriented Gradients
UdeC	Universidad de Concecpión
RoI	Region of Interest
SGD	Stochastic Gradient Descent

## References

[1] Manz J., Nsoga J., Diazenza J.B., Sita S., Bakana G.M.B., Francois A., Ndomou M., Gouado I., Mamonekene V. (2023). Nutritional composition, heavy metal contents and lipid quality of five marine fish species from Cameroon coast. Heliyon.

[2] Food and Agriculture Organization of the United Nations (2018). The Status of World Fisheries and Aquaculture. Meeting the Sustainable Development Goals.

[3] Nahuelhual L., Saavedra G., Blanco G., Wesselink E., Campos G., Vergara X. (2018). On super fishers and black capture: Images of illegal fishing in artisanal fisheries of southern Chile. Mar. Policy.

[4] Gunnar S., Rosenberg A.A. (2005). Combining control measures for more effective management of fisheries under uncertainty: Quotas, effort limitation and protected areas. Philos. Trans. R. Soc. London. Ser. Biol. Sci..

[5] Ye Y., Gutierrez N.L. (2017). Ending fishery overexploitation by expanding from local successes to globalized solutions. Nat. Ecol. Evol..

[6] Urban P., Bekkevold D., Degel H., Hansen B., Jacobsen M., Nielsen A., Nielsen E. (2023). Scaling from eDNA to biomass: Controlling allometric relationships improves precision in bycatch estimation. ICES J. Mar. Sci..

[7] SERNAPESCA SERNAPESCA Informes de Gestión. http://www.sernapesca.cl/informes/resultados-gestion.

[8] Beaudreau A.H., Levin P.S., Norman K.C. (2011). Using folk taxonomies to understand stakeholder perceptions for species conservation. Conserv. Lett..

[9] Rojo M.M., Noronha T.D. (2016). Low-technology industries and regional innovation systems: The salmon industry in Chile. J. Spat. Organ. Dyn..

[10] Plotnek E., Paredes F., Galvez M., Pérez-Ramírez M. (2016). From unsustainability to MSC certification: A case study of the artisanal Chilean South Pacific hake fishery. Rev. Fish. Sci. Aquac..

[11] Schaap R.J., Gonzalez-Poblete E., Aedo K.L.S., Diekert F. (2022). Risk, Restrictive Quotas, and Income Smoothing.

[12] Fischer J. (2014). Fish Identification Tools for Biodiversity and Fisheries Assessments: Review and Guidance for Decision-Makers.

[13] Bendall C., Hiebert S., Mueller G. (1999). Experiments in Situ Fish Recognition Systems Using Fish Spectral and Spatial Signatures. https://pubs.usgs.gov/publication/ofr99104.

[14] Hossain E., Alam S.M.S., Ali A.A., Amin M.A. Fish activity tracking and species identification in underwater video. Proceedings of the 2016 5th International Conference on Informatics, Electronics and Vision (ICIEV).

[15] Spampinato C., Giordano D., Di Salvo R., Chen-Burger Y.H.J., Fisher R.B., Nadarajan G. Automatic fish classification for underwater species behavior understanding. Proceedings of the 1st ACM International Workshop on Analysis and Retrieval of Tracked Events and Motion in Imagery Streams, ARTEMIS ’10.

[16] Hu J., Li D., Duan Q., Han Y., Chen G., Si X. (2012). Fish Species Classification by Color, Texture and Multi-class Support Vector Machine Using Computer Vision. Comput. Electron. Agric..

[17] White D., Svellingen C., Strachan N. (2006). Automated measurement of species and length of fish by computer vision. Fish. Res..

[18] Storbeck F., Daan B. (2001). Fish species recognition using computer vision and a neural network. Fish. Res..

[19] Cheng J.H., Sun D.W. (2014). Hyperspectral imaging as an effective tool for quality analysis and control of fish and other seafoods: Current research and potential applications. Trends Food Sci. Technol..

[20] Cheng J.H., Qu J.H., Sun D.W., Zeng X.A. (2014). Visible/near-infrared hyperspectral imaging prediction of textural firmness of grass carp (ctenopharyngodon idella) as affected by frozen storage. Food Res. Int..

[21] Sivertsen A.H., Heia K., Hindberg K., Godtliebsen F. (2012). Automatic nematode detection in cod fillets (*Gadus morhua* L.) by hyperspectral imaging. J. Food Eng..

[22] Costa C., Antonucci F., Menesatti P., Pallottino F., Boglione C., Cataudella S. (2013). An advanced colour calibration method for fish freshness assessment: A comparison between standard and passive refrigeration modalities. Food Bioprocess Technol..

[23] Ramírez D., Pezoa J.E., LeVan P.D., Wijewarnasuriya P., D’Souza A.I. (2018). Spectral vision system for discriminating small pelagic species caught by small-scale fishing. Proceedings of the Infrared Sensors, Devices, and Applications VIII.

[24] Rathi D., Jain S., Indu S. Underwater fish species classification using convolutional neural network and deep learning. Proceedings of the 2017 9th International Conference on Advances in Pattern Recognition (ICAPR).

[25] Deep B.V., Dash R. Underwater fish species recognition using deep learning techniques. Proceedings of the 2019 6th International Conference on Signal Processing and Integrated Networks (SPIN).

[26] Ulucan O., Karakaya D., Turkan M. A large-scale dataset for fish segmentation and classification. Proceedings of the 2020 Innovations in Intelligent Systems and Applications Conference (ASYU).

[27] Alsmadi M.K., Almarashdeh I. (2022). A survey on fish classification techniques. J. King Saud-Univ.-Comput. Inf. Sci..

[28] Deka J., Laskar S., Baklial B. (2023). Automated Freshwater Fish Species Classification using Deep CNN. J. Inst. Eng. India Ser. B.

[29] Song H., Yang W.W., Dai S., Du L., Sun Y.C. (2020). Using dual-channel CNN to classify hyperspectral image based on spatial-spectral information. Math. Biosci. Eng..

[30] Chen L., Wei Z., Xu Y. (2020). A lightweight spectral–spatial feature extraction and fusion network for hyperspectral image classification. Remote. Sens..

[31] Jiang J., Liu D., Gu J., Süsstrunk S. What is the space of spectral sensitivity functions for digital color cameras?. Proceedings of the 2013 IEEE Workshop on Applications of Computer Vision (WACV).

[32] Liu Y., Pu H., Sun D.W. (2021). Efficient extraction of deep image features using convolutional neural network (CNN) for applications in detecting and analysing complex food matrices. Trends Food Sci. Technol..

[33] Naseri M.N., Agrawal A.P. Impact of transfer learning on siamese networks for face recognition with few images per class. Proceedings of the 2021 Asian Conference on Innovation in Technology (ASIANCON).

[34] Siri C.S. Enhancing cartoon recognition in real time: Comparative analysis of CNN, ResNet50, and VGG16 deep learning models. Proceedings of the 2023 2nd International Conference on Augmented Intelligence and Sustainable Systems (ICAISS).

[35] Soares L., Botelho S., Nagel R., Drews P.L. A visual inspection proposal to identify corrosion levels in marine vessels using a deep neural network. Proceedings of the 2021 Latin American Robotics Symposium (LARS), 2021 Brazilian Symposium on Robotics (SBR), and 2021 Workshop on Robotics in Education (WRE).

[36] Mujtaba D.F., Mahapatra N.R. A study of feature importance in fish species prediction neural networks. Proceedings of the 2022 International Conference on Computational Science and Computational Intelligence (CSCI).

[37] Mujtaba D.F., Mahapatra N.R. Fish species classification with data augmentation. Proceedings of the 2021 International Conference on Computational Science and Computational Intelligence (CSCI).

[38] Yin C., Zhu Y., Liu S., Fei J., Zhang H. (2020). Enhancing network intrusion detection classifiers using supervised adversarial training. J. Supercomput..

[39] Ben Tamou A., Benzinou A., Nasreddine K. (2022). Targeted data augmentation and hierarchical classification with deep learning for fish species identification in underwater images. J. Imaging.

[40] Tripathy S., Singh R. (2022). Convolutional neural network: An overview and application in image classification. Proceedings of the 3rd International Conference on Sustainable Computing: SUSCOM 2021.

[41] Ahmed F., Basak B., Chakraborty S., Karmokar T., Reza A.W., Imam O.T., Arefin M.S. (2022). Developing a classification CNN model to classify different types of fish. Proceedings of the Intelligent Computing & Optimization: Proceedings of the 5th International Conference on Intelligent Computing and Optimization 2022 (ICO2022).

[42] Zhang P., He J., Huang W., Zhang J., Yuan Y., Chen B., Yang Z., Xiao Y., Yuan Y., Wu C. (2023). Water Pipeline Leak Detection Based on a Pseudo-Siamese Convolutional Neural Network: Integrating Handcrafted Features and Deep Representations. Water.

[43] Guo X., Zhao X., Liu Y., Li D. (2019). Underwater sea cucumber identification via deep residual networks. Inf. Process. Agric..

[44] Garbin C., Zhu X., Marques O. (2020). Dropout vs. batch normalization: An empirical study of their impact to deep learning. Multimed. Tools Appl..

[45] Jose J.A., Kumar C.S., Sureshkumar S. (2022). Tuna classification using super learner ensemble of region-based CNN-grouped 2D-LBP models. Inf. Process. Agric..

[46] Salazar J.J., Garland L., Ochoa J., Pyrcz M.J. (2022). Fair train-test split in machine learning: Mitigating spatial autocorrelation for improved prediction accuracy. J. Pet. Sci. Eng..

[47] Ahmed M.A., Hossain M.S., Rahman W., Uddin A.H., Islam M.T. (2023). An advanced Bangladeshi local fish classification system based on the combination of deep learning and the internet of things (IoT). J. Agric. Food Res..

[48] Chicco D., Jurman G. (2020). The advantages of the Matthews correlation coefficient (MCC) over F1 score and accuracy in binary classification evaluation. BMC Genom..

[49] Chen S., Webb G.I., Liu L., Ma X. (2020). A novel selective naïve Bayes algorithm. Knowl.-Based Syst..

[50] Chauhan V.K., Dahiya K., Sharma A. (2019). Problem formulations and solvers in linear SVM: A review. Artif. Intell. Rev..

[51] Pant N., Bal B.K. Improving Nepali ocr performance by using hybrid recognition approaches. Proceedings of the 2016 7th International Conference on Information, Intelligence, Systems & Applications (IISA).

[52] Nugroho K.A. A comparison of handcrafted and deep neural network feature extraction for classifying optical coherence tomography (OCT) images. Proceedings of the 2018 2nd International Conference on Informatics and Computational Sciences (ICICoS).

[53] Kernbach J.M., Staartjes V.E. (2022). Foundations of machine learning-based clinical prediction modeling: Part II—Generalization and overfitting. Mach. Learn. Clin. Neurosci. Found. Appl..

